# Recognition of ErbB Family Dimers by the EGF-like Domain of NRG1alpha and Beta: Implications for Ligand-Based CAR Therapy

**DOI:** 10.3390/ijms27156813

**Published:** 2026-07-29

**Authors:** Alex Novikov, Alon Naumchik, Rajashri Banerji, Yariv Greenshpan, Kamran Waidha, Baisali Bhattacharya, Moshe Elkabets, Olga Radinsky, Angel Porgador

**Affiliations:** The Shraga Segal Department of Microbiology, Immunology and Genetics, Faculty of Health Sciences, Ben-Gurion University of the Negev, Beer-Sheva 8410501, Israel

**Keywords:** CAR-T therapy, HER2, NRG1, immunotherapy, 3D tumor microenvironment, NK92 cells

## Abstract

HER-positive cancers comprise a heterogeneous group of malignancies driven by dysregulated activation of the human epidermal growth factor receptor (HER/ERBB) family. While chimeric antigen receptor (CAR) T-cell therapies targeting HER2 have demonstrated potent anti-tumor capabilities, their clinical translation remains hampered by safety hurdles and antigen escape. We developed a modular “Targeted Chimeric Artificial Reporter” (TcAR) system using the natural epidermal growth factor (EGF)-like binding domains of neuregulin-1 (NRG1) isoforms to decode complex HER dimerization profiles. Leveraging the superior targeting plasticity of the β-isoform, we engineered NRG1β-based CAR-T and CAR-natural killer (NK) cells. Our study demonstrates that NRG1β-directed therapy overcomes therapeutic antigen escape in HER2-depleted models and exerts potent anti-tumor activity within complex three-dimensional (3D) tissue microenvironments ex vivo, exhibiting a significantly safer, muted inflammatory profile compared to clinical standards.

## 1. Introduction

HER-positive cancers comprise a heterogeneous group of malignancies driven by dysregulated activation of the human epidermal growth factor receptor (HER/ERBB) family. This family includes four transmembrane receptor tyrosine kinases: EGFR (HER1), HER2, HER3, and HER4 [1]. These receptors regulate signaling pathways that control cell proliferation, survival, differentiation, and migration. Activation occurs predominantly through homo- or heterodimer formation, whereby ligand binding induces conformational changes that promote receptor pairing and subsequent transphosphorylation [2,3]. Within this network, HER1 binds multiple ligands and participates broadly in signaling; HER2 lacks a known direct ligand and serves as a preferred dimerization partner that amplifies signaling output; and HER3, despite having impaired kinase activity, functions as a potent allosteric activator. The HER2–HER3 heterodimer is considered one of the most potent oncogenic signaling units within the ErbB family [3,4]. While historically associated with breast cancer, the clinical significance of HER family dysregulation extends to numerous solid tumors. HER3 upregulation and altered HER-family signaling are implicated in the pathogenesis and therapy resistance of colorectal cancer [5,6,7], gastric cancer [8,9,10], and non-small cell lung cancer (NSCLC) [4,11,12]. Current precision oncology strategies, such as monoclonal antibodies (e.g., Trastuzumab) and Tyrosine Kinase Inhibitors (TKIs), have markedly improved the treatment of HER-family-driven cancers. Nevertheless, therapeutic resistance remains a major limitation and is often mediated by adaptive rewiring of receptor signaling, including compensatory HER3 upregulation and enhanced HER2-HER3 heterodimerization [13,14]. This rewiring can sustain downstream pro-survival signaling, particularly phosphoinositide 3-kinase (PI3K)/protein kinase B (AKT) activation, thereby reducing the efficacy of HER2-targeted monotherapy [15].

The complexity of this network is further modulated by ligands such as Neuregulin-1 (NRG1). The *NRG1* gene encodes a family of alternatively spliced growth factor isoforms, including α and β variants [16,17]. All NRG1 isoforms contain an EGF-like domain required for receptor binding, but the α and β forms differ in receptor affinity, signaling potency, and tissue distribution [18]. In general, NRG1β isoforms act as higher-affinity ligands for ERBB receptors, particularly HER3 and HER4, whereas NRG1α isoforms display distinct functional properties across tissue types [17,18]. Biochemical evaluations demonstrate that the isolated NRG1β EGF-like domain exhibits a highly potent, low-nanomolar basal inhibitory concentration (IC_50_) of approximately ~10^−8^ M toward the ErbB3 receptor interface, driving a 10- to 100-fold higher binding affinity compared to the α-variant [19]. Crucially, while neither NRG1 isoform binds HER2 directly, their binding to HER3 or HER4 drives the formation of potent heterodimers with HER2 (e.g., HER2-HER3), thereby initiating robust intracellular signaling cascades like PI3K/AKT and mitogen-activated protein kinase (MAPK) pathways [20,21]. Despite the availability of HER-targeted monoclonal antibodies and TKIs, durable responses are frequently thwarted by tumor heterogeneity and adaptive resistance mechanisms [22,23]. In addition, although HER3-directed antibodies and antibody-drug conjugates have shown clinical promise, no HER3-targeted monoclonal antibody has yet received Food and Drug Administration (FDA) approval [24,25,26]. A primary route of therapeutic failure is target antigen escape [27,28], wherein tumors dynamically downregulate HER2 expression to evade immune destruction, rendering HER2-centric therapies ineffective [29,30]. Furthermore, while Chimeric Antigen Receptor (CAR) T-cell therapies targeting HER2 have demonstrated potent anti-tumor capabilities [31], their clinical translation for solid tumors remains severely hampered by two critical safety hurdles: on-target/off-tumor toxicity against healthy tissues expressing basal HER2 levels [32,33], and the propensity to induce severe, hyper-inflammatory complications such as Cytokine Release Syndrome (CRS) [34,35]. Thus, there is an urgent unmet need for engineered cellular therapies capable of sustaining robust efficacy against tumors with low or shifting HER2 profiles, while maintaining a strictly controlled safety and cytokine release profile.

A strategy of employing ERBB ligands instead of anti-ERBB Abs as the targeting moiety of anti-ERBB CARs should be thus investigated. This strategy is also conceptually aligned with the emerging principle of affinity-tuned CAR design, in which reduced or physiologically constrained target engagement may improve discrimination between tumor cells with high target density and normal tissues with lower physiological expression [36,37]. Such tuning may be particularly important for solid-tumor antigens, where most targets are tumor-associated rather than strictly tumor-specific. Therefore, ligand-based CAR architecture provides a biologically relevant platform, with physiological affinities as compared to high-affinity monoclonal antibodies (mAbs), that can be further tuned by ligand truncation, mutation, spacer design, or CAR-expression level to balance antitumor activity with safety.

To address these critical translational challenges, we developed a modular “Targeted Chimeric Artificial Reporter” (TcAR) system. This dual-purpose platform could serve both as a functional diagnostic tool and a therapeutic scaffold; it utilizes the natural EGF-like binding domains of the NRG1α and NRG1β isoforms to systematically decode complex HER dimerization profiles, circumventing the limitations of standard expression-based diagnostics. By exploiting the distinct thermodynamic binding specificities of these isoforms, we first mapped their capacity to differentiate between endogenous HER complexes. Subsequently, leveraging the superior targeting versatility of the β-isoform identified in our reporter screens, we engineered NRG1β-based CAR-T and CAR-NK92 cells. We systematically benchmarked this ligand-based approach against a standard clinical anti-HER2 scFv [38]. Our study demonstrates that the NRG1β-directed cellular therapy could overcome therapeutic challenges associated with target antigen downregulation and loss in HER2-depleted tumor models but also exerts potent anti-tumor activity within complex 3D tissue microenvironments ex vivo, all while exhibiting a significantly safer, attenuated inflammatory profile compared to current clinical standards.

## 2. Results

### 2.1. Design and Generation of Ligand-Based Artificial Reporters for HER Family Detection

To develop a tool for decoding the complex dimerization patterns of HER family receptors, we engineered a panel of chimeric artificial reporters expressed in murine thymoma BW5147 cells. This cell line was selected as a “clean” background due to its lack of endogenous HER receptor expression. The reporter constructs were designed incorporating the EGF-like domains of human NRG1α and NRG1β, which serve as the primary natural ligands for HER3 (ErbB3). 

**Figure 1:**
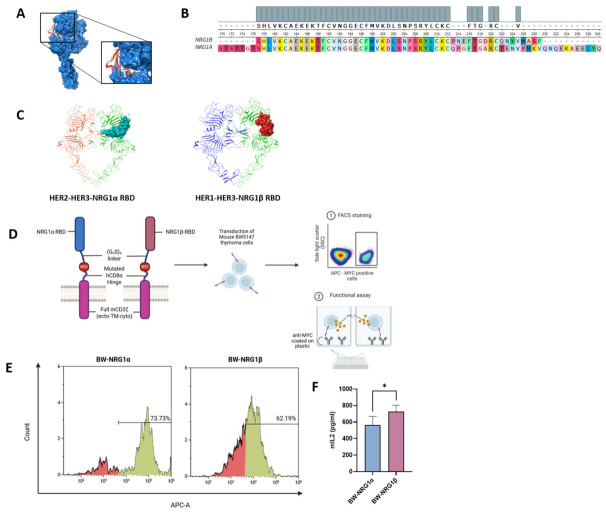
Design, functionality, and validation of BW-based reporters targeting HER family receptors. (**A**) Superimposed structures of various reported NRG1β-HER complexes. (**B**) Pairwise sequence alignment of NRG1β and NRG1α isoforms recognizing the corresponding 53-amino acid sequence. (**C**) Molecular docking complexes of the selected NRG1α and NRG1β receptor binding domains (RBDs). Representative 3D molecular docking models illustrate the 53-amino acid EGF-like domains bound to the structural interfaces of HER2-HER3 and HER1-HER3 heterodimers. Corresponding center energy scores for these interactions, as well as for the HER3-HER3 homodimer, are summarized in Table 1. (**D**) Schematic of NRG1α and NRG1β constructs, featuring ligand domains, MYC tags, a cysteine-mutated human CD8α hinge and a consolidated full-length mCD3ζ signaling chain (ecto-TM-cyto). BW5147 cells were transduced to generate functional reporters. (**E**) Fluorescence-activated cell sorting (FACS) analysis for APC-MYC-positive BW-reporter cells. In the histograms, the green shaded regions represent the positively transduced cell populations expressing the constructs, whereas the red shaded regions indicate the negative background populations. This confirms the successful transduction and expression of the reporter constructs in thymoma cells. (**F**) Murine interleukin-2 (mIL-2) secretion upon anti-MYC stimulation. Both reporters demonstrated robust activation, with BW-NRG1β exhibiting a moderately higher response than BW-NRG1α. * *p* < 0.05 (Student’s *t*-test, mean ± SEM, *n* = 5).

While various lengths of the NRG1 EGF-like domain have been previously described in the literature to retain biological activity [39], we sought to identify a minimal, optimal sequence capable of manifesting differential binding affinities toward specific HER3 homo- and heterodimers. To this end, we analyzed multiple reported X-ray Crystallographic structures of NRG1β bound to different HER receptors, leading to the identification of a 53-amino acid sequence (Figure 1A). Pairwise sequence alignment was further used to identify the corresponding NRG1α amino acid sequence (Figure 1B). 

A refined 53-amino acid sequence corresponding to residues 177–229 of the NRG1 isoforms exhibited highly favorable binding behaviors and distinct differential affinities. Specifically, as summarized in Table 1, docking against the HER1-HER3 heterodimer yielded center energy scores of −765.7 and −915.4 for the α and β sequences, respectively. For the HER2-HER3 heterodimer, both sequences demonstrated similarly robust recognition, with lowest energy scores of −771.4 for α and −721.7 for β. Interestingly, docking energy scores indicated that the 53-amino acid sequence of the β isoform recognized the HER3-HER3 homodimer with higher thermodynamic stability compared to the α isoform (−748.8 vs. −712.1, respectively). 

**Figure A1:**
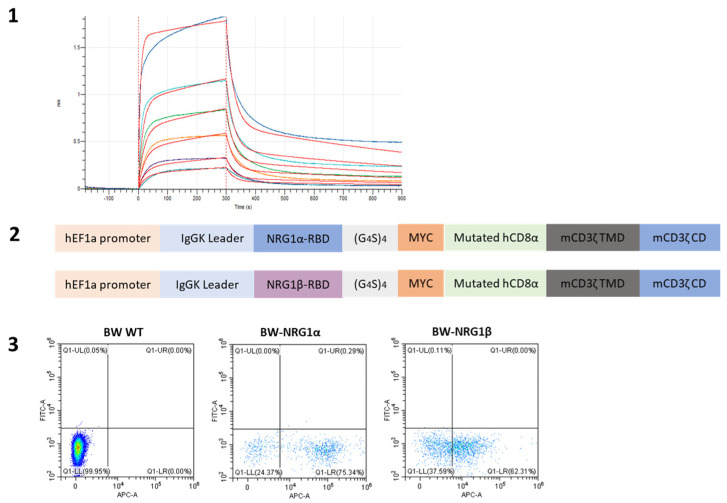
(**1**) BLI analysis of NRG1β-Ig binding kinetics to HER3. Sensograms depict the association (0–300 s) and dissociation (300–900 s) of His-HER3 at various concentrations (ranging from 15.6 to 1000 nM via two-fold serial dilutions, including a 0 nM reference control) to NRG1β-Ig (3 µg/mL) immobilized on anti-human IgG Fc Capture (AHC2) biosensors. Colored lines represent experimental data; red lines indicate the global fit to a two-state model (KD = 128 nM, R^2^ = 0.988). (**2**) Linear schematic of the reporter gene cassettes. The constructs are driven by a human EF1α (hEF1α) promoter and feature an IgGκ leader sequence to direct membrane trafficking, followed by the NRG1α or NRG1β ligand domain, a flexible (G4S)4 linker, a MYC tag, a cysteine-mutated hCD8α hinge, and the mCD3ζ transmembrane (TMD) and cytoplasmic (CD) signaling domains. (**3**) Representative flow cytometry dot plots showing the raw data for APC-anti-MYC surface staining of the transduced BW5147 cells.

Consequently, based on these optimal center energy scores, we selected these precise 53-amino acid segments to serve as the functional targeting heads for our system. We hereafter refer to these sequences as NRG1α-RBD and NRG1β-RBD (Receptor Binding Domains) (Figure 1C,D, Table 1 and Figure A1(2)).

To quantitatively validate these computational predictions, we expressed a 53-amino-acid fragment of NRG1β in an IgG-like fusion format (termed NRG1β-Ig) and performed kinetic binding assays using Bio-Layer Interferometry (BLI) against the recombinant His-HER3 receptor. The calculated equilibrium dissociation constant (K_D_) was approximately 128 nM (Figure A1(1)).

To facilitate both robust detection and functional readout of binding to ErbB3-2-1 homo/heterodimers, we established BW-based reporters based on modularly designed chimeric receptors (Figure 1D). Each reporter construct features an N-terminal ligand-binding domain (NRG1α-RBD or NRG1β-RBD) connected via a flexible (G4S)4 linker to a MYC-tag, which facilitates routine monitoring of surface expression by flow cytometry. To provide optimal structural flexibility while preventing unwanted spontaneous homodimerization, this extracellular targeting module was anchored to the cell surface utilizing a cysteine-mutated human CD8α hinge region. Finally, to drive intracellular signal transduction, the hinge was fused to the full-length murine CD3ζ chain (comprising the ectodomain, transmembrane, and cytoplasmic regions). This comprehensive modular architecture ensures that specific engagement with HER dimers effectively triggers BW-cell activation, culminating in measurable murine interleukin-2 (mIL-2) secretion.

Following retroviral transduction, we first validated the structural design of the chimeric receptors by assessing surface expression. Using the incorporated MYC-tag, flow cytometry analysis confirmed high and stable expression levels for both reporter lines (BW-NRG1α and BW-NRG1β), demonstrating the efficiency of the constructs to mediate membrane-associated expression of the chimeric receptors (Figure 1E).

Subsequently, to verify the signaling capability of the CD3ζ domain, we stimulated the cells with plate-bound anti-MYC antibodies. Both reporters responded with significant murine IL-2 secretion (Figure 1F). While BW-NRG1β exhibited a modestly higher response to uniform anti-MYC crosslinking compared to BW-NRG1α, both constructs demonstrated robust and highly significant activation. Crucially, this validates that the intracellular signaling machinery is fully functional in both reporters, establishing them as a reliable platform to evaluate the specific binding readouts of the NRG1-RBDs against distinct ErbB homo- and heterodimers in subsequent assays.

### 2.2. Functional Validation of Differential HER Dimer Recognition by BW-Reporters

**Figure 2:**
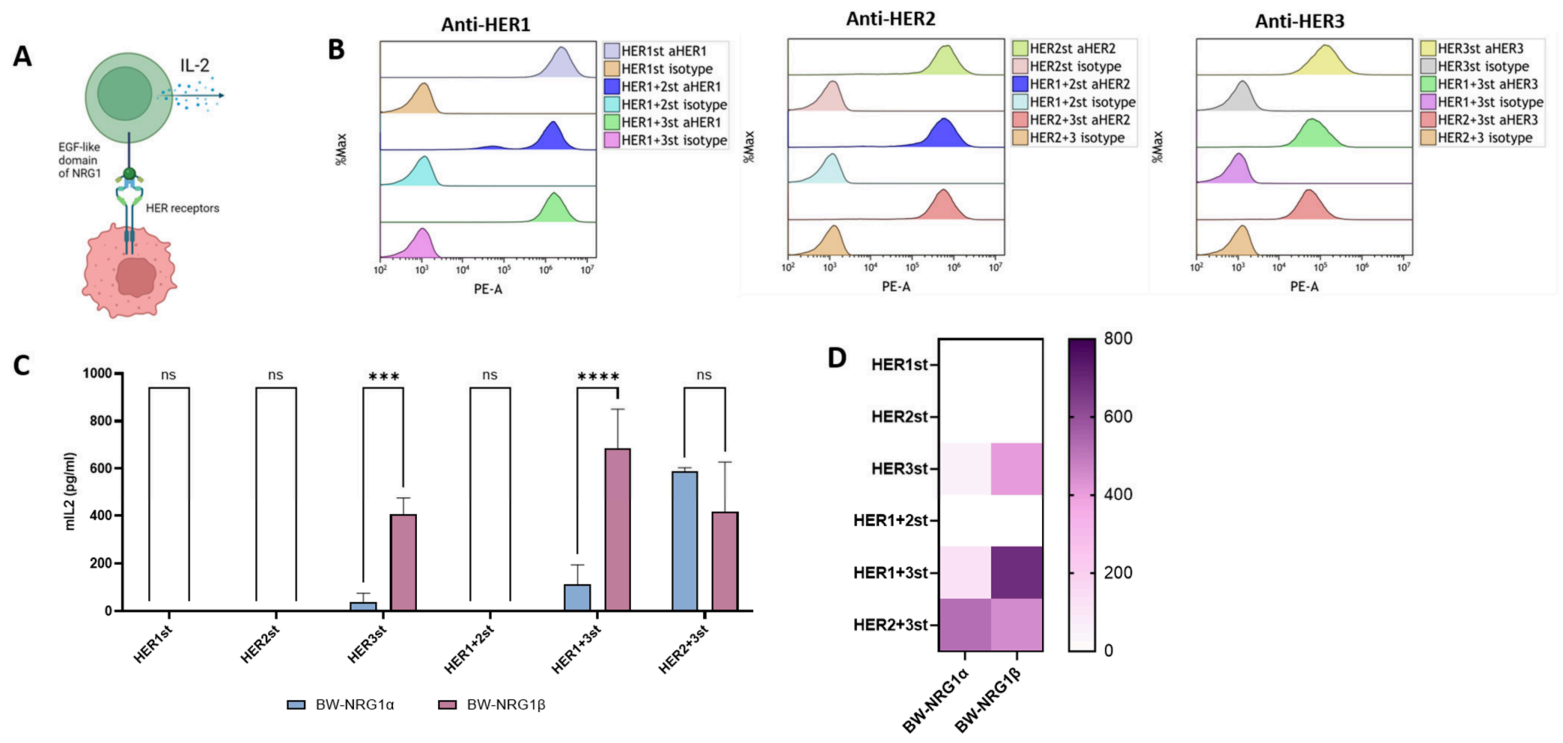
Mechanism of BW-reporter function, HER receptor expression profiles, and reporter functionality across HER standards. (**A**) Schematic representation of the BW-reporter mechanism. The EGF-like domain of NRG1 binds to HER receptors on target cells, resulting in IL-2 secretion by the BW-reporters. (**B**) Flow cytometry analysis of HER receptor expression in HER1, HER2, and HER3 standards. Histograms represent receptor-specific PE-labeled antibody staining using anti-HER1, anti-HER2 and anti-HER3. (**C**) IL-2 secretion by BW-reporters co-cultured with HER standards measured by ELISA. Data show reporter-specific responses to HER1, HER2, HER3 homodimers (HER1st, HER2st, HER3st), and HER heterodimers (HER1+2st, HER1+3st, HER2+3st). Bars represent the mean ± SEM from three independent experiments (*n* = 3). ns, not significant, *** *p* < 0.001, **** *p* < 0.0001 (Unpaired Student’s *t*-test was used for comparisons within each HER group). (**D**) Heatmap representation of the mean mIL-2 secretion data shown in panel (**C**).

**Figure A2:**
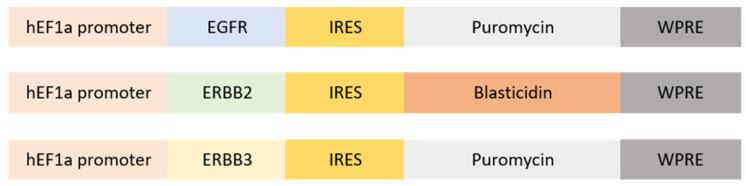
Linear schematics of the pHAGE2 lentiviral vectors encoding full-length human HER1 (EGFR), HER2 (ERBB2), and HER3 (ERBB3). Receptor expression is driven by the human EF1α (hEF1α) promoter. An internal ribosome entry site (IRES) enables the bi-cistronic expression of the respective antibiotic resistance genes (puromycin or blasti-cidin) for stable cell selection, followed by a Woodchuck Hepatitis Virus Posttranscrip-tional Regulatory Element (WPRE) to enhance transgene stability and expression.

To evaluate the capacity of the engineered BW-reporters to discriminate between distinct HER receptor complexes, we established an in vitro co-culture assay (Figure 2A). First, we generated a comprehensive panel of target cells expressing specific HER homo- and heterodimers, serving as controlled ‘HER standards’. To this end, murine BW5147 cells were transduced with pHAGE2 lentiviral vectors encoding full-length human HER1, HER2, or HER3, linked to puromycin or blasticidin resistance cassettes (Figure A2). Homodimer-expressing standards were generated via single transductions, whereas heterodimer-expressing targets (HER1+2, HER1+3, HER2+3) were established through simultaneous co-transduction. Flow cytometry analysis confirmed the precise surface expression profiles of the respective HER receptors across these cell lines, validating their utility as defined target standards (Figure 2B). 

Following the validation of the target standards, we co-cultured the BW-NRG1α and BW-NRG1β reporters with these cells and quantified murine IL-2 secretion to assess functional receptor engagement. Demonstrating the absolute specificity of the engineered targeting modules, neither reporter exhibited activation when stimulated with cells expressing only HER1 (HER1st), HER2 (HER2st), or the HER1/HER2 heterodimer (HER1+2st) (Figure 2C). This confirms that signaling is strictly dependent on the presence of HER3 on the target cells.

Strikingly, the reporters exhibited distinct functional profiles when challenged with HER3-containing complexes. Upon co-culture with the HER3 homodimer standard (HER3st), the BW-NRG1β reporter demonstrated robust activation, whereas the BW-NRG1α reporter showed minimal baseline signaling. A similarly significant trend was observed against the HER1/HER3 heterodimer (HER1+3st), with BW-NRG1β secreting significantly higher levels of IL-2 compared to BW-NRG1α (Figure 2C). These functional results elegantly mirror our in silico thermodynamic predictions, confirming the superior binding capacity of the β-isoform to these specific complexes.

Conversely, when engaged with the highly potent HER2/HER3 heterodimer standard (HER2+3st), the activation landscape shifted dramatically. Both reporters triggered robust IL-2 secretion; however, interestingly, BW-NRG1α demonstrated a strong tendency towards higher activation levels compared to BW-NRG1β, although this trend did not reach statistical significance in this assay configuration (Figure 2C). This profound enhancement highlights the unique functional dynamics of the HER2/HER3 complex, which fully unleashes the signaling capacity of the α-isoform. These distinct activation signatures, comprehensively summarized in the functional heatmap (Figure 2D), demonstrate that the modular BW-reporter system successfully translates differential binding affinities into specific cellular readouts, enabling the precise decoding of HER dimerization states on target cells.

### 2.3. Translating Reporter Specificities to Endogenous HER Profiles on Human Cell Lines

**Figure 3:**
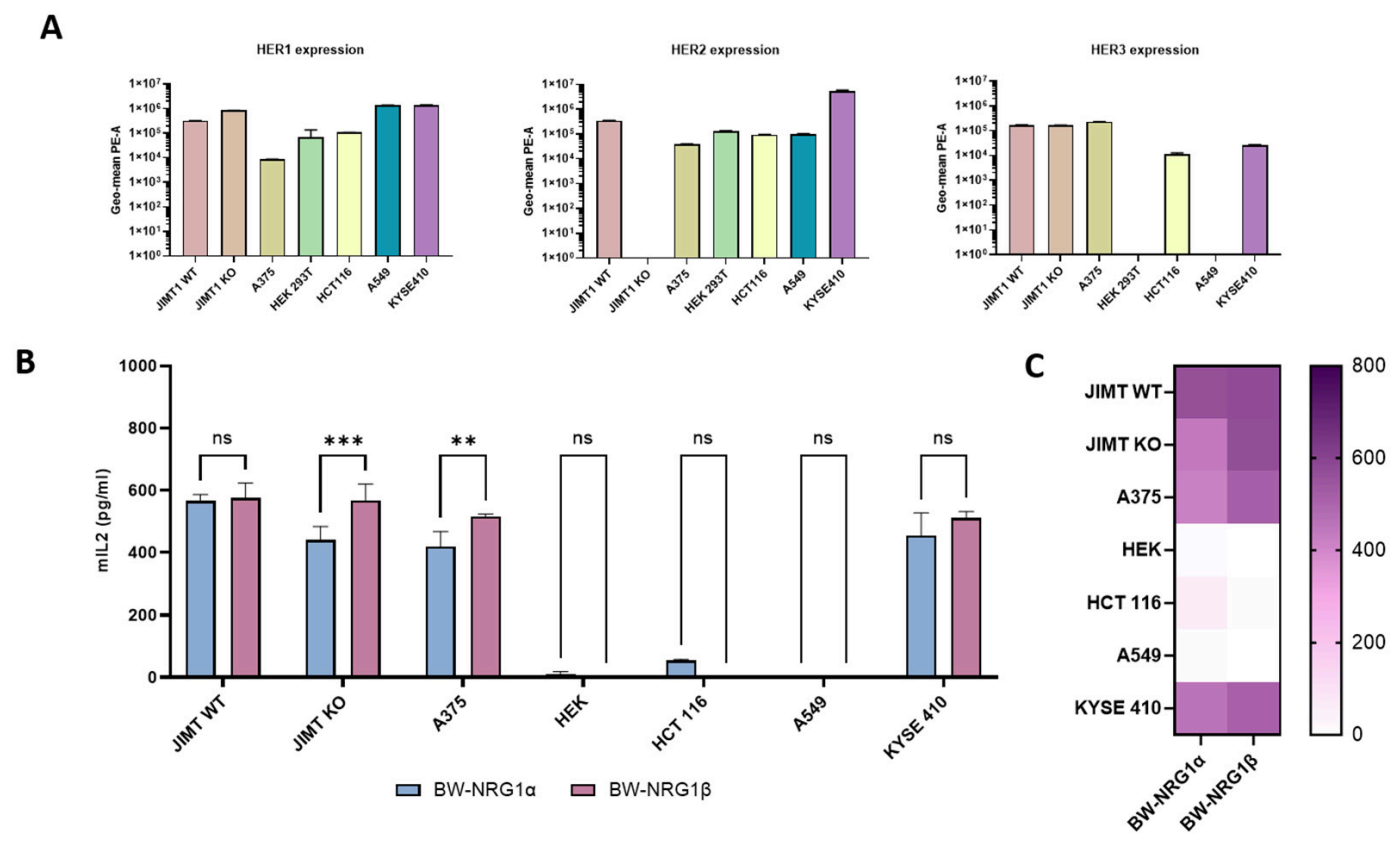
HER expression profiles and reporter functionality across a panel of human cell lines. (**A**) Surface expression of HER1, HER2, and HER3 on the indicated cell lines (JIMT1 WT, JIMT1 KO, A375, HEK 293T, HCT116, A549, and KYSE410) assessed by flow cytometry. Bars represent the geometric mean fluorescence intensity (Geo-mean PE-A) of receptor-specific staining using anti-HER1, anti-HER2, and anti-HER3. (**B**) mIL-2 secretion by BW-NRG1α and BW-NRG1β reporters co-cultured with the cell lines, measured by ELISA. Bars represent the mean ± SEM from three independent experiments (*n* = 3). ns, not significant, ** *p* < 0.01, *** *p* < 0.001 (Unpaired Student’s *t*-test was used for comparisons within each cell line). (**C**) Heatmap representation of the mean IL-2 secretion data shown in panel (**B**).

To determine whether the binding rules established using engineered standards apply to physiological, endogenously expressed HER networks, we expanded our co-culture assay to a diverse panel of human cell lines. We first quantified the baseline surface expression of HER1, HER2, and HER3 across these lines (Figure 3A). This profiling revealed a wide spectrum of endogenous HER signatures, ranging from cells lacking HER3 (A549, HEK 293T) to those overexpressing specific receptors, such as predominant HER1 (JIMT HER2-knockout), HER2 (KYSE 410, JIMT1 WT) and HER3 (A375).

Upon co-culture, the functional readouts of the BW-reporters adequately mirrored the endogenous expression landscapes (Figure 3B,C). Reinforcing the absolute requirement for HER3 engagement, target cells with negligible HER3 expression (A549 and HEK 293T) failed to trigger any detectable IL-2 secretion from either reporter, regardless of their HER1 or HER2 status. Furthermore, HCT116 cells, which exhibit very low endogenous expression across all three receptors, also failed to elicit a robust response.

Crucially, the differential activation profiles of the α and β isoforms observed on the synthetic standards were faithfully reproduced on these physiological targets. When encountering cells expressing HER3 but lacking high levels of HER2—such as A375 and the JIMT1 KO line—BW-NRG1β significantly outperformed BW-NRG1α, demonstrating its superior capacity to engage HER3-centric complexes independent of HER2. The JIMT1 isogenic pair serves as a particularly elegant proof of concept: genetic ablation of HER2 in the JIMT1 KO cells resulted in a striking reduction in BW-NRG1α activation, whereas BW-NRG1β maintained a robust signaling output.

Conversely, on cell lines displaying high endogenous levels of both HER2 and HER3, such as KYSE410 and JIMT1 WT, the signaling gap between the isoforms was bridged. Both BW-NRG1α and BW-NRG1β were potently activated to comparable levels on these targets, corroborating our earlier finding that the HER2/HER3 heterodimer serves as an exceptionally potent and universally recognized signaling hub for both NRG1 variants. Collectively, these data demonstrate that while both isoforms effectively target HER2/HER3-overexpressing cells, the NRG1β targeting module exhibits a significantly broader recognition profile, maintaining robust functionality even against tumors with limited HER2 expression. Given this superior targeting plasticity, we selected the β-isoform as our lead candidate to be engineered into fully functional Chimeric Antigen Receptors (CARs) for subsequent evaluation in therapeutic effector cells.

### 2.4. Engineering and Functional Profiling of NRG1β-Based CAR-T Cells Against HER2-Variant Tumors

**Figure 4:**
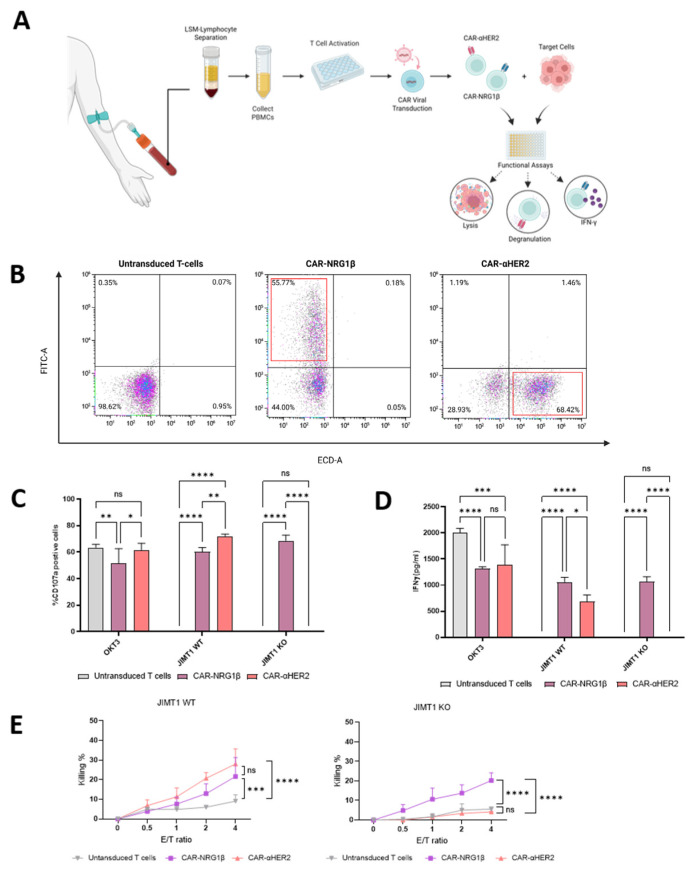
Design and in vitro functional characterization of NRG1β-based and anti-HER2 CAR-T cells. (**A**) Schematic representation of the experimental workflow, including primary human T cell isolation, lentiviral transduction to generate CAR-T cells, and subsequent in vitro functional assays. (**B**) Representative flow cytometry plots confirming CAR expression on engineered primary human T cells compared to untransduced T cells (CAR-NRG1β is tracked by ZsGreen on the FITC axis, and CAR-aHER2 is tracked by mCherry on the ECD axis). (**C**) CD107a degranulation assay evaluating CAR-T cell activation following a 4 h co-culture with JIMT1 WT and JIMT1 KO target cells. The percentage of CD107a+ T cells was determined by flow cytometry. Plate-bound OKT3 was used as a positive control for T cell activation. (**D**) IFNγ secretion by CAR-T cells following co-culture with JIMT1 WT and JIMT1 KO targets, or OKT3 stimulation, measured by ELISA. (**E**) In vitro cytotoxicity assay demonstrating the specific lysis of JIMT1 WT (**left**) and JIMT1 KO (**right**) tumor cells. Target cells were pre-labeled with Vybrant DiD and co-cultured with the indicated CAR-T cells for 3 h at varying effector-to-target (E:T) ratios. Specific lysis was determined by flow cytometry based on DAPI uptake within the DiD-positive population. All bar graphs and killing curves represent the mean ± SEM of independent experiments (*n* = 3). Statistical significance was determined using a Two-way ANOVA followed by Tukey’s multiple comparisons test. ns = not significant, * *p* < 0.05, ** *p* < 0.01, *** *p* < 0.001, **** *p* < 0.0001.

Building on the superior targeting plasticity of the β-isoform observed in our reporter assays, we engineered primary human T cells to express a second-generation CAR incorporating the NRG1β ligand domain. To benchmark our design against current clinical standards, we generated a parallel CAR utilizing a well-characterized anti-HER2 single-chain variable fragment (scFv) derived from the clinical antibody trastuzumab (CAR-aHER2). Both receptors featured a CD28 costimulatory domain and a CD3ζ activation domain (Figure 4A). Flow cytometry analysis confirmed robust and comparable surface expression of both CAR constructs on primary human T cells (Figure 4B).

**Figure A3:**
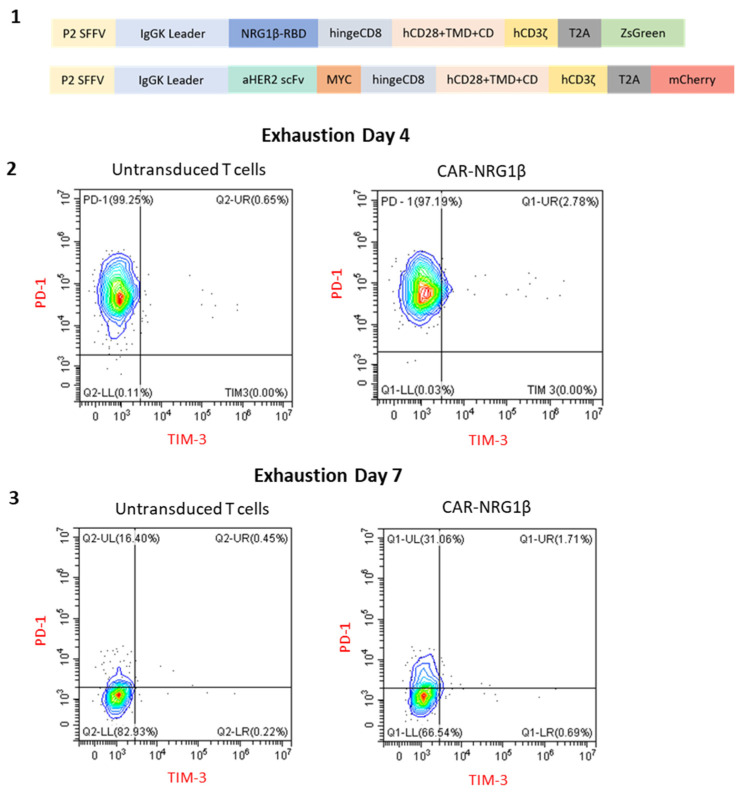
(**1**) Schematic representation of the lentiviral constructs encoding CAR-NRG1β and the clinical benchmark CAR-aHER2. The constructs are driven by an SFFV promoter and comprise an IgGκ leader sequence, the respective targeting domain (NRG1β or an anti-HER2 single-chain variable fragment (scFv) derived from trastuzumab with a MYC tag), a CD8α hinge, a CD28 transmembrane and costimulatory domain, and a CD3ζ activation domain. A T2A ribosomal skip sequence links the CAR to a fluorescent reporter (ZsGreen or mCherry) to track transduction efficiency. (**2**) Phenotypic analysis of T cells on Day 4 post-activation (prior to target engagement). Flow cytometry contour plots show the surface expression of PD-1 (*y*-axis) and TIM-3 (*x*-axis) on untransduced control T cells (**left**) and CAR-NRG1β T cells (**right**). (**3**) Phenotypic analysis of T cells on Day 7 post-activation during the expansion phase. Flow cytometry contour plots show the surface expression of PD-1 (*y*-axis) and TIM-3 (*x*-axis) on untransduced control T cells (**left**) and CAR-NRG1β T cells (**right**).

Importantly, the integration of the natural NRG1β targeting domain did not induce spontaneous, antigen-independent tonic signaling. Phenotypic analysis of the engineered T cells during the expansion phase (Days 4 and 7 post-activation), prior to target engagement, revealed no upregulation of the deep exhaustion marker TIM-3 compared to untransduced control cells (Figure A3(2,3)). Both populations exhibited dynamic clearance of the activation marker PD-1, which was highly expressed on Day 4 following acute anti-CD3/IL-2 manufacturing activation and subsequently downregulated by Day 7. This resolution of transient activation markers, coupled with the persistent absence of TIM-3, confirms the cells maintain a robust, non-exhausted profile prior to tumor encounter.

To evaluate the effector functions of the engineered T cells, we co-cultured them with the isogenic JIMT1 tumor cell models. Upon engagement with JIMT1 WT cells, both CAR-NRG1β and CAR-aHER2 T cells exhibited potent activation, demonstrating high levels of CD107a degranulation (Figure 4C) and robust cytolytic activity across multiple effector-to-target ratios (Figure 4E, left). Notably, while both CARs effectively killed the wild-type tumor cells, CAR- NRG1β triggered a mildly higher secretion of IFN-γ compared to CAR-aHER2 (Figure 4D).

The critical advantage of the NRG1β-based CAR became evident when challenging the T cells with the HER2-ablated JIMT1 KO cells, mimicking a scenario of therapeutic antigen escape. As expected, the clinical benchmark CAR-aHER2 completely failed to recognize the JIMT1 KO targets, resulting in a total loss of IFN-γ secretion (Figure 4D) and an inability to mount any cytolytic response above baseline untransduced T cells (Figure 4E, right). In stark contrast, CAR-NRG1β T cells fully retained their effector capabilities, demonstrating significant CD107a degranulation (Figure 4C), robust IFN-γ production (Figure 4D), and highly effective target cell lysis (Figure 4E, right). These results underscore the unique therapeutic potential of the CAR-NRG1β design: it not only matches the cytolytic efficacy of clinical HER2-CARs against high-antigen tumors but also provides a vital rescue strategy against tumors that have downregulated HER2 expression.

### 2.5. CAR-NRG1β Exhibits Potent Ex Vivo Anti-Tumor Efficacy with a Favorable, Muted Cytokine Profile

To evaluate the therapeutic potential of the CAR-NRG1β cells in a more complex tumor setting, we assessed their ex vivo efficacy and safety profile against 3D JIMT1 WT tumor tissue explants utilizing the established Tumor Ex Vivo Analysis (TEVA) platform [40]. These explants were preserved in formalin-fixed paraffin-embedded (FFPE) blocks and organized into Tissue Microarrays (TMAs) to allow for standardized high-throughput analysis of the tumor microenvironment. Tumor cell proliferation was quantified in 5 µm sections using immunohistochemistry (IHC) staining for the proliferation marker Ki67, following a 16–18 h primary antibody incubation to ensure optimal antigen recognition.

**Figure 5:**
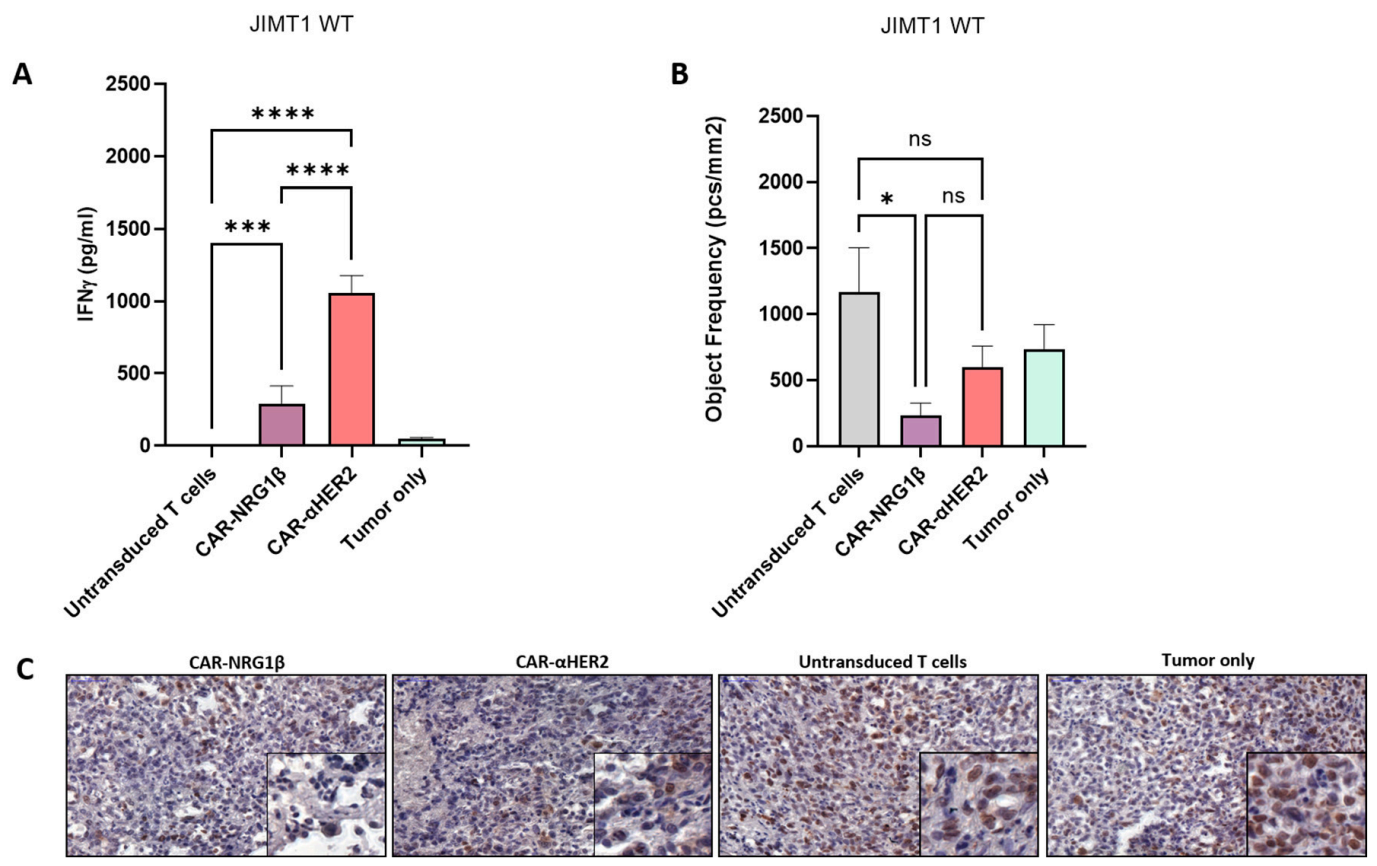
Ex vivo efficacy, proliferation inhibition, and cytokine secretion profile of CAR-NRG1β T cells. (**A**) IFN-γ secretion levels. Effector T cells (Untransduced, CAR-NRG1β, or CAR-αHER2) were co-cultured overnight with JIMT1 WT tumor slices, and supernatants were analyzed by ELISA. Bars represent mean ± SEM. Statistical significance was determined using ordinary one-way ANOVA followed by Tukey’s multiple comparisons test. (**B**) Quantification of tumor cell proliferation assessed by Ki67 object frequency (pieces/mm^2^). Statistical significance was determined using a Kruskal–Wallis test followed by Dunn’s multiple comparisons test comparing all treatment groups. Bars represent mean ± SEM. (**C**) Representative immunohistochemistry (IHC) images of TMA (Tissue Microarrays) sections stained for the proliferation marker Ki67 (brown) and counterstained with hematoxylin (blue). Images were acquired at 40× magnification. Scale bars = 50 µm. Insets display digitally magnified fields. ns = not significant, * *p* < 0.05, *** *p* < 0.001, **** *p* < 0.0001.

Notably, only CAR-NRG1β treatment resulted in a statistically significant reduction in Ki67+ proliferating tumor cells compared to Untransduced T cells or untreated tumor controls (*p* < 0.05; Figure 5B,C). While the clinical benchmark CAR-αHER2 showed a downward trend in proliferation, it failed to reach statistical significance against the control in this 3D model (*p* > 0.99). Importantly, the anti-proliferative capacity of CAR-NRG1β was statistically comparable to that of CAR-αHER2 (*p* = 0.38), demonstrating equivalent therapeutic potency.

A major limitation of current CAR-T therapies in solid tumors is the induction of severe toxicities, such as Cytokine Release Syndrome (CRS), driven by excessive pro-inflammatory cytokine secretion. To evaluate this risk, we measured IFN-γ levels in the co-culture supernatants. Strikingly, while CAR-αHER2 cells secreted massive amounts of IFN-γ, indicating a hyper-inflammatory response, CAR-NRG1β cells exhibited a significantly lower, muted cytokine release profile (Figure 5A). Taken together, these data demonstrate that the ligand-based CAR-NRG1β provides potent anti-tumor efficacy equivalent to the clinical standard, but with a substantially safer and tightly regulated immunological profile.

### 2.6. NRG1β-Based CAR Architecture Provides a Versatile Platform for NK92 Cell Engineering

**Figure 6:**
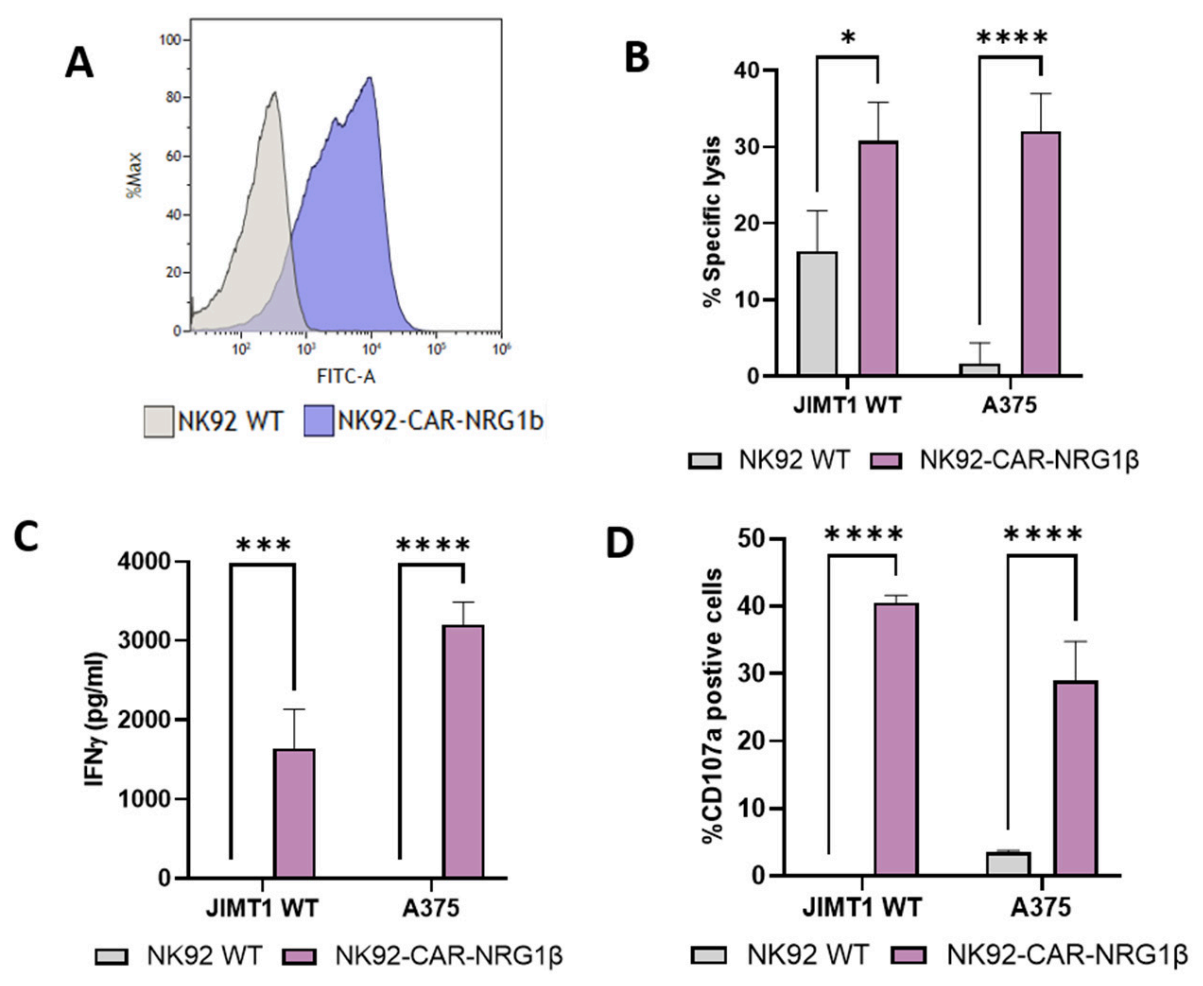
Generation and functional validation of NRG1β-based CAR-NK92 cells. (**A**) Representative flow cytometry histogram confirming successful CAR expression on engineered human NK92 cells (purple) compared to parental NK92 WT cells (grey). CAR expression was tracked via a fluorescent reporter on the FITC axis. (**B**) In vitro cytotoxicity of NK92 cells against JIMT1 WT and A375 tumor cells. NK92 WT (grey) or NK92-CAR-NRG1β (purple) cells were co-cultured with target cells for 3 h at an E:T ratio of 3:1. Target cells were pre-labeled with vibrant-DiD, and specific lysis was determined by flow cytometry based on DAPI uptake within the DiD-positive population. (**C**) IFN-γ secretion levels. NK92 cells were co-cultured with the indicated target cells for 16 h. Supernatants were subsequently collected and analyzed by ELISA to quantify cytokine production. (**D**) Degranulation assay. The percentage of CD107a+ NK92 cells was determined by flow cytometry following a 4 h co-culture with target cells. Bars represent the ± SEM of three independent experiments (*n* = 3). Statistical significance was determined using a Two-way ANOVA followed by Šídák’s multiple comparisons test. * *p* < 0.05, *** *p* < 0.001, **** *p* < 0.0001.

To demonstrate the modularity and broad applicability of the NRG1β targeting domain, we extended our platform to Natural Killer (NK) cells by engineering a CAR-NK92 cell line. First, successful lentiviral transduction and robust CAR expression on NK92 cells were confirmed by flow cytometry (Figure 6A). The functional potency of these NRG1β-CAR-NK92 cells was then evaluated against JIMT1 WT cells and the melanoma-derived A375 cell line, which serves as a model for alternative HER-family expression profiles.

In short-term cytotoxicity assays (3 h, E:T 3:1), NRG1β-CAR-NK92 cells demonstrated significantly enhanced specific lysis compared to parental NK92 WT cells across both target lines (Figure 6B). Notably, while NK92 WT cells exhibited negligible baseline activity against A375 cells, the introduction of the NRG1β-CAR induced a robust killing response, reaching levels comparable to those observed in the JIMT1 WT model. This redirected cytotoxicity was closely associated with potent effector cell activation, as evidenced by a dramatic increase in CD107a degranulation (Figure 6D) and high levels of IFN-γ secretion following 16 h co-culture (Figure 6C). Consistent with the killing data, CAR-mediated activation was particularly pronounced in the A375 model, confirming that the NRG1β domain effectively redirects NK92 cells toward diverse tumor targets. Collectively, these results validate the NRG1β-based CAR as a versatile and potent tool for the development of next-generation “off-the-shelf” NK-cell-based immunotherapies.

## 3. Discussion

The development of the TcAR platform represents a significant shift from traditional protein expression-based diagnostics toward a functional, dimerization-centric approach for targeting HER-family-driven malignancies. By leveraging the natural thermodynamic specificities of NRG1 isoforms, we demonstrated that ligand-based reporters can systematically decode the complex receptor landscapes of tumor cells, providing a more accurate reflection of their oncogenic signaling state than standard IHC or FISH techniques. This functional mapping proved instrumental in identifying the NRG1β isoform as a superior targeting domain, characterized by its remarkable plasticity in recognizing both HER3 homodimers and HER2-HER3 heterodimers. This structural flexibility is particularly critical in the context of therapeutic antigen escape, a primary driver of resistance in HER2-targeted therapies. Our results show that while clinical-standard scFv-based CARs lose efficacy upon HER2 downregulation, the NRG1β-based CAR-T cells maintain potent cytotoxicity by engaging alternative HER dimerization partners. This ability to “follow” the tumor’s adaptive rewiring of receptor signaling suggests that ligand-based architectures could provide more durable responses in heterogeneous solid tumors.

Another advantage is that although the HER3 ligand-based CAR might be less potent in vitro as compared to the anti-HER2 CAR, this should not necessarily be interpreted as a disadvantage. Rather, it may reflect a deliberate and potentially favorable trade-off between maximal short-term cytotoxicity and improved selectivity. High-affinity CARs can respond to low antigen densities and therefore may provide stronger in vitro killing, but this same property can increase the risk of on-target/off-tumor recognition when the antigen is also expressed at physiological levels in normal tissues. By contrast, affinity-tuned or ligand-based CARs may require higher receptor density, more favorable receptor conformation, or productive receptor clustering, thereby preferentially responding to tumor cells in which HER3 is overexpressed or functionally engaged in oncogenic ERBB signaling. This concept is supported by recent studies showing that affinity-tuned CARs can reduce off-tumor toxicity while preserving antitumor efficacy, including HER2, CAIX, ICAM-1, and mesothelin CAR models [36,41]. Therefore, the lower apparent potency of the HER3 ligand-based CAR in vitro may represent a wider therapeutic window in vivo, particularly for a target such as HER3, where safety and tumor-versus-normal discrimination are central considerations [37,41].

Beyond its efficacy in antigen-depleted models, a key translational advantage of the NRG1β-CAR system is the observed decoupling of anti-tumor activity from excessive inflammatory signaling. While the clinical benchmark anti-HER2 CAR induced massive IFN-γ secretion—a hallmark of potential Cytokine Release Syndrome (CRS)—our ligand-derived CAR achieved equivalent tumor killing and proliferation inhibition with a significantly modulated cytokine profile. Mechanistically, this phenomenon highlights a critical functional divergence between physical cytotoxicity and cytokine hyper-secretion. High-affinity synthetic scFvs often drive supra-physiological immunological synapses that enforce massive, sustained transcriptional bursts of pro-inflammatory cytokines. In contrast, the natural ligand-receptor affinity of the NRG1β-HER3 interaction is functionally sufficient to trigger targeted granule exocytosis (perforin/granzyme release) for robust serial killing, yet it falls below the rigid activation threshold required to perpetuate extreme IFN-γ release. This uncoupling of direct cytolysis from systemic inflammatory signaling represents a substantial safety advantage for solid tumor targeting.

Notably, in the complex 3D tissue microenvironment model, CAR-NRG1β demonstrated a more robust anti-proliferative effect, being the only construct to reach statistical significance against the control group. This suggests that natural ligand-receptor interactions may provide a more “physiological” and tightly regulated activation signal compared to the high-affinity, often supra-physiological binding of synthetic scFvs, potentially leading to more reliable therapeutic responses in dense tumor architectures. Furthermore, the successful integration of this targeting domain into both CAR-T and CAR-NK92 platforms underscores the modularity and versatility of the TcAR-derived sequences. By demonstrating robust efficacy across different effector cell types and within complex 3D tissue microenvironments ex vivo, this study establishes a framework for next-generation cellular therapies that are not only resistant to target loss but also exhibit a broadened therapeutic window. Future clinical translation will necessitate evaluating these systems in patient-derived xenograft models to further validate their safety and long-term persistence in the face of a dynamic tumor microenvironment.

## 4. Materials and Methods

### 4.1. Homology Modelling and Molecular Docking

Structures for NRG1-RBD, HER1-HER3 and HER2-HER3 were modelled using the ExPASy Swiss-Model web server (https://swissmodel.expasy.org/ (accessed on 19 July 2026)). Structural docking simulations for the HER3 homodimer and HER-NRG1 complexes were generated using the Cluspro2.0 docking mode. The corresponding cluster center energy scores (*E*) were calculated from the formula:

*E* = 0.40*E*_rep_ − 0.40*E*_att_ + 600*E*_elec_ + 1.00*E*_DARS_

*E*_rep_ and *E*_att_ represent the repulsive and attractive van der Waals energies, *E*_elec_ is the electrostatic term, and *E*_DARS_ is a structure-based potential derived from the decoys as reference state (DARS) method.

### 4.2. Sequence Alignment

The Sequence alignment for NRG1β-RBD and NRG1α isoform was performed using the Clustal Omega webserver (https://www.ebi.ac.uk/jdispatcher/msa/clustalo (accessed on 19 July 2026)). Alignment visualization was conducted using Unipro UGENE v53.1.

### 4.3. Bio-Layer Interferometry (BLI) Binding Assays

All affinity measurements were performed on an Octet R8e instrument (Sartorius, Göttingen, Germany) using anti-human IgG Fc Capture (AHC2) biosensors (cat. no. 18-5142). Assays were conducted at 25 °C in kinetic buffer (1× KB) consisting of 1× PBS, 0.002% (*v*/*v*) Tween-20, and 0.01% (*w*/*v*) BSA. Prior to the experiment, biosensors were hydrated in double-distilled water (ddH_2_O) for 10 min. Samples were loaded into a 384-well plate (Greiner, cat. no. 781209, Frickenhausen, Germany) at a final volume of 80 µL per well. The plate was maintained inside the instrument at 25 °C with orbital shaking at 1000 rpm. The biosensors were loaded with 3 µg/mL of NRG1β-Ig for 400 s, washed in 1× KB for 60 s, and incubated in fresh 1× KB for 180 s to establish a baseline. The ligand-loaded sensors were then immersed in wells containing His-HER3 at various concentrations (ranging from 15.6 to 1000 nM via two-fold serial dilutions, using 0 nM as a reference control) for 300 s to measure association, followed by dissociation in 1× KB for 600 s. Kinetic parameters were analysed using Octet^®^ Analysis Studio software (version 13.1.0.38; Sartorius) and fit to a two-state binding model. A coefficient of determination (R^2^) greater than 0.95 was used as the threshold for a valid statistical fit. The interaction yielded an equilibrium dissociation constant (KD) of 128 nM under the two-state model, with an R^2^ of 0.988.

### 4.4. Cell Lines and Culture Media

The murine thymoma cell line BW5147 was utilized as the parental line for generating both the artificial reporter system and the standard cell lines. These cells were cultured in RPMI 1640 medium (Gibco, Grand Island, NY, USA) supplemented with 10% fetal bovine serum (FBS), 1 mM sodium pyruvate, 2 mM L-glutamine, 10 mM HEPES, 0.1 mM non-essential amino acids (NEAA), and 1% Penicillin-Streptomycin (all from Sartorius/Gibco).

All human-established cell lines utilized in this research (including the JIMT-1 breast cancer target cell model and the functional effector/reporter platforms) are commercial and standardized lines. They were originally obtained from recognized international biological resource centers, specifically the American Type Culture Collection (ATCC) and the German Collection of Microorganisms and Cell Cultures (DSMZ).

The Human tumor cell lines, including JIMT1 (wild-type and HER2-KO), FaDu (hypopharyngeal carcinoma), A375 (melanoma), HCT116 (colorectal carcinoma), A549 (lung carcinoma), KYSE410 (esophageal squamous cell carcinoma), and HEK293T (for viral production), were maintained in DMEM (Gibco) supplemented with 10% FBS, HEPES, sodium pyruvate, Pen-Strept, L-glutamine, and NEAA. For effector cells, primary human T cells were cultured in Nutri-T medium (Sartorius) supplemented with 400 U/mL of recombinant human IL-2 (rhIL-2). The NK92 cell line was cultured in Alpha MEM (Gibco) supplemented with 12.5% FBS, 12.5% Horse Serum, 0.2 mM inositol, 0.1 mM 2-mercaptoethanol, 0.02 mM folic acid, and 120 U/mL rhIL-2 (PeproTech, Cranbury, NJ, USA). All cells were maintained at 37 °C in a humidified atmosphere containing 5% CO_2_.

### 4.5. Cloning and Construct Design

Three distinct types of viral constructs were engineered for this study:

1. Ligand-based Reporters (for BW5147): The EGF-like domains of human NRG1α and NRG1β (53 amino acids) were cloned into the pHAGE2 lentiviral vector under the control of the hEF1α promoter. These constructs were designed to express an N-terminal IgGκ leader sequence, the specific ligand/scFv domain, a flexible (G4S)4 linker, a Myc-tag for surface detection, a human CD8 hinge, and the transmembrane and cytoplasmic signaling domains of murine CD3ζ (mCD3ζ).
2. HER Standards (for BW5147): To generate system calibration standards, sequences encoding full-length human HER1, HER2, and HER3 were cloned into the pHAGE2 vector.
3. CAR Constructs (for T cells and NK92): For therapeutic application, the ligand-binding domains (NRG1α, NRG1β or anti-HER2 scFv) were cloned into a pSFFV promoter-based vector. These second-generation CAR constructs consisted of the ligand/scFv domain, a (G4S)4 linker, and the human CD28 hinge, transmembrane, and costimulatory domains, fused to the human CD3ζ signaling domain. To facilitate sorting and tracking, the constructs were linked via a T2A self-cleaving peptide to a fluorescent reporter (ZsGreen for ligands, mCherry for anti-HER2).

### 4.6. Viral Production and Transduction

Viral particles were produced in HEK293T cells following transfection with the expression vector and packaging plasmids using JetPrime^®^ reagent (Polyplus, New York, NY, USA). Supernatants containing viral particles were collected 48 h post-transfection, centrifuged (500× *g*, 5 min), and filtered. 

Reporter and Standard Cells: BW5147 cells were transduced with the viral supernatant and selected using Puromycin (10 µg/mL) or Blasticidin (10 µg/mL). To generate “Standard” cells expressing specific HER heterodimers (e.g., HER1 + HER2, HER1 + HER3, HER2 + HER3), BW5147 cells were co-transduced simultaneously with the respective viral vectors. Homodimer-expressing standards were generated by single transduction. Surface expression was verified by flow cytometry using the following antibodies: PE-anti-HER1 (IgG1, κ; clone AY13, BioLegend, San Diego, CA, USA), PE-anti-HER2 (IgG1, κ; clone 24D2, BioLegend), and PE-anti-HER3 (IgG2a, κ; clone 1B4C3, BioLegend). The corresponding isotype controls used were PE-mouse IgG1, κ (clone MOPC-21, BioLegend) and PE-mouse IgG2a, κ (clone MOPC-173, BioLegend). Primary T Cells and NK92: Peripheral blood mononuclear cells (PBMCs) were isolated from healthy donor blood by density gradient centrifugation using LSM-Lymphocyte Separation Media (MP Biomedicals, Santa Ana, CA, USA). T cells were activated with Ultra-LEAF anti-human CD3 (clone OKT3 Cat# 317347, 100 ng/mL) and rhIL-2 (100 U/mL) for 24 h prior to transduction. T cells and NK92 cells were transduced with the CAR viral vectors in the presence of BX795 (6 nM) and Rosuvastatin (5 nM) to enhance efficiency, as previously described [42]. Transduced cells were expanded in their respective media supplemented with 300 U/mL rhIL-2 (for T cells) or 100 U/mL (for NK92).

### 4.7. Flow Cytometry and Surface Expression Analysis

To verify surface expression of the chimeric receptors, transduced cells (5 × 10^4^/well) were stained with an anti-Myc antibody (Clone MABE282, BioLegend) followed by an APC-conjugated goat anti-mouse IgG + IgM secondary antibody (Jackson ImmunoResearch, West Grove, PA, USA, Cat# 115-136-068). HER receptor expression profiles on tumor cell lines were analyzed using fluorophore-conjugated anti-HER1, anti-HER2 and anti-HER3 antibodies. Viability was assessed using DAPI staining (1 μg/mL). Data were acquired using a CytoFLEX flow cytometer (Beckman Coulter, Brea, CA, USA) and analyzed using CytExpert software v2.5.

For baseline exhaustion phenotyping, engineered and untransduced T cells (5 × 10^4^ cells/well) were collected on days 4 and 7 post-activation. Cells were washed and incubated for 30 min in the dark with the following fluorophore-conjugated antibodies at a 1:500 dilution: PE-Cy7 anti-human CD3 (clone UCHT1, Cat# 300420), PE anti-human CD279/PD-1 (clone EH122H7, Cat# 329906), and Brilliant Violet 421™ anti-human CD366/TIM-3 (clone F38-2E2, Cat# 345008), all purchased from BioLegend. Following primary staining, cells were washed and stained with 7-AAD Viability Staining Solution (1:1000 dilution, Cat# 420403, BioLegend) to exclude dead cells. Data acquisition and analysis were performed as described above.

### 4.8. Functional Reporter Assay (IL-2 ELISA)

The specific activation of BW-reporter cells was assessed by measuring murine IL-2 (mIL-2) secretion. Reporter cells (5 × 10^4^/well) were co-cultured with target tumor cells or engineered BW-standards (1 × 10^5^/well) in 96-well plates for 16–18 h. For positive controls, reporter cells were stimulated with plate-bound anti-Myc antibody (2.5 µg/mL). Supernatants were collected, and mIL-2 levels were quantified using a standard sandwich ELISA kit (Thermo Fisher, Waltham, MA, USA) according to the manufacturer’s instructions.

### 4.9. CAR-T/NK Cytotoxicity and Activation Assays

Degranulation Assay: CAR-T or CAR-NK cells (5 × 10^4^) were co-cultured with target cells (1.5 × 10^5^) in the presence of APC-conjugated anti-CD107a antibody (Clone H4A3, BioLegend) for 4 h. Cells were then washed, stained for surface markers (CD3 for T cells or CD56 for NK cells), and analyzed by flow cytometry. 

IFN-γ Secretion: Effector cells were co-cultured with target cells at a 1:2 ratio for 16–18 h. Human IFN-γ levels in the supernatant were quantified by ELISA. 

Killing Assay: Target cells were labeled with Vybrant™ DiD cell-labeling solution (Cat#V22887, Invitrogen, Carlsbad, CA, USA) and co-cultured with CAR-T cells at varying Effector:Target (E:T) ratios (0.5:1, 1:1, 2:1) for 3 h. Cell death was quantified by flow cytometry based on DAPI uptake by DiD-positive cells.

### 4.10. Ex Vivo Tumor Explant Assay (Tumor Ex Vivo Analysis—TEVA)

**Figure A4:**
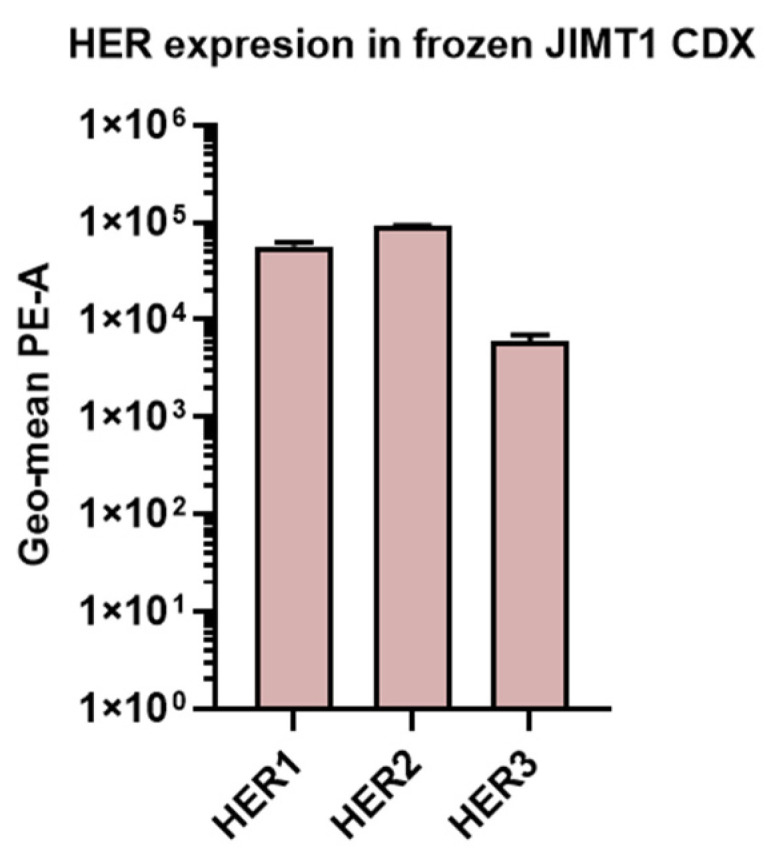
Surface expression levels of HER1, HER2, and HER3 receptors on cells dissociated from frozen JIMT1 wild-type (WT) cell line-derived xenograft (CDX) tumors. Receptor expression was quantified by flow cytometry using specific PE-conjugated antibodies. Data are presented as the geometric mean fluorescence intensity (Geo-mean PE-A). Bars represent the mean ± SEM from two biological replicates (*n* = 2).

Human breast cancer cell line-derived xenografts (JIMT-1 CDXs) with endogenous HER expression profiles (Figure A4) were established in vivo. Tumor ex vivo analysis (TEVA) was performed as previously described [40], by aseptically excising fresh, live tumor masses from mice and sectioning them into viable 3D tissue explants of approximately 2 × 2 × 2 mm^3^. These live explants were placed in 48-well tissue culture plates and incubated with 20K of CAR-T cells expressing NRG1β, or anti-HER2 under sterile conditions at 37 °C, 95% relative humidity, and 5% CO_2_ in a CO_2_ incubator. Untransduced T cells served as the negative control. Following the incubation period, culture supernatants were harvested for IFN-γ secretion analysis via ELISA, and the treated tumor explants were immediately collected for downstream histological processing.

### 4.11. FFPE Block Preparation and Tissue Microarray (TMA) Construction

Following the completion of the live ex vivo treatment phase, the harvested tumor tissue explants were fixed in 4% paraformaldehyde and embedded in paraffin (FFPE) using an automated tissue-processing machine (Leica Biosystems, Nußloch, Germany). Subsequently, using 3 mm T-Sue^tm^ punch needles (Simport, Beloeil, QC, Canada), tissue microarray (TMA) blocks containing a maximum of 24 tissue explants were constructed from the tumor tissue paraffin blocks.

### 4.12. Histological Analysis: Ki67 Immunohistochemistry Staining

Tissue sections of 5 μm thickness were cut from the constructed TMA blocks using a fully automated rotary microtome (Leica RM2255, Nussloch, Germany). For immunohistochemistry (IHC) staining, the sections were first deparaffinized using xylene and rehydrated through a graded ethanol series. Heat-mediated antigen retrieval was conducted by immersing the sections in citrate buffer (pH 6.0) at 95 °C for 30 min. To counteract endogenous peroxidase activity, a 3% hydrogen peroxide (H_2_O_2_) solution was applied to the sections for 20 min, followed by a thorough rinse. Sections were then blocked at room temperature for 1 h using a blocking solution comprising phosphate-buffered saline (PBS) with 0.1% Tween and 5% bovine serum albumin (BSA). The sections were incubated overnight at 4 °C with a Ki67 anti-human primary antibody (Merck, Darmstadt, Germany, Ref: 275R-14) diluted at 1:200 according to the manufacturer’s guidelines. Following primary antibody incubation, an IHC ABC kit (VECTASTAIN, Cat: VE-PK-6200, Vector Laboratories, Inc., Newark, CA, USA) was employed for enzymatic color detection. The sections were counterstained with hematoxylin to enable visualization of cellular nuclei and mounted using VectaMount (Cat# H-5000, Vector Laboratories, Inc., Newark, CA, USA) permanent mounting medium. High-resolution digital images of the stained slides were acquired using a Pannoramic Scanner (3DHISTECH, Budapest, Hungary) and analyzed through Pannoramic Viewer 1.15.4 software to assess and quantify the immunohistochemical staining patterns.

### 4.13. Statistical Analysis

Data are presented as mean ± Standard Error of the Mean (SEM). Statistical significance was determined using one-way ANOVA followed by Tukey’s post-hoc test for multiple comparisons, or Student’s *t*-test for pairwise comparisons, using GraphPad Prism 9 software. *p*-values < 0.05 were considered statistically significant.

## Figures and Tables

**Table 1:** ClusPro 2.0 centre energies of different docked NRG1-HER complexes.

Receptor Complex	NRG1α Score	NRG1β Score
HER3-HER3 Homodimer	−712.1	−748.8
HER1-HER3 Heterodimer	−765.7	−915.4
HER2-HER3 Heterodimer	−771.4	−721.7

## Data Availability

The raw data supporting the conclusions of this article will be made available by the authors on request.

## Abbreviations

The following abbreviations are used in this manuscript:

HER/ERBB	Human epidermal growth factor receptor
EGFR	Epidermal growth factor receptor
NSCLC	Non-small cell lung cancer
TKIs	Tyrosine kinase inhibitors
PI3K/AKT	Phosphoinositide 3-kinase/Protein kinase B
NRG1	Neuregulin-1
EGF	Epidermal growth factor
MAPK	Mitogen-activated protein kinase
CAR	Chimeric antigen receptor
CRS	Cytokine release syndrome
TcAR	Targeted chimeric artificial reporter
RBD	Receptor binding domain
FFPE	Formalin-fixed paraffin-embedded
TMA	Tissue microarray
IHC	Immunohistochemistry
FBS	Fetal bovine serum
NEAA	Non-essential amino acids
rhIL-2	Recombinant human interleukin-2
mIL-2	Murine interleukin-2
PBMC	Peripheral blood mononuclear cells
ELISA	Enzyme-linked immunosorbent assay
SEM	Standard error of the mean
NSG	NOD-scid IL2Rγnull (mice)
DAPI	4′,6-diamidino-2-phenylindole
E:T	Effector-to-target ratio
IVIS	In vivo imaging system
